# Transcriptional and Linkage Analyses Identify Loci that Mediate the Differential Macrophage Response to Inflammatory Stimuli and Infection

**DOI:** 10.1371/journal.pgen.1005619

**Published:** 2015-10-28

**Authors:** Musa A. Hassan, Kirk D. Jensen, Vincent Butty, Kenneth Hu, Erwan Boedec, Pjotr Prins, Jeroen P. J. Saeij

**Affiliations:** 1 Wellcome Trust Centre for Molecular Parasitology, University of Glasgow, Glasgow, United Kingdom; 2 Department of Biology, Massachusetts Institute of Technology, Cambridge, Massachusetts, United States of America; 3 School of Biotechnology, University of Strasbourg, Strasbourg, France; 4 Laboratory of Nematology, Wageningen University, Wageningen, The Netherlands; 5 Department of Pathology, Microbiology & Immunology, University of California, Davis, Davis, California, United States of America; University of Chicago, UNITED STATES

## Abstract

Macrophages display flexible activation states that range between pro-inflammatory (classical activation) and anti-inflammatory (alternative activation). These macrophage polarization states contribute to a variety of organismal phenotypes such as tissue remodeling and susceptibility to infectious and inflammatory diseases. Several macrophage- or immune-related genes have been shown to modulate infectious and inflammatory disease pathogenesis. However, the potential role that differences in macrophage activation phenotypes play in modulating differences in susceptibility to infectious and inflammatory disease is just emerging. We integrated transcriptional profiling and linkage analyses to determine the genetic basis for the differential murine macrophage response to inflammatory stimuli and to infection with the obligate intracellular parasite *Toxoplasma gondii*. We show that specific transcriptional programs, defined by distinct genomic loci, modulate macrophage activation phenotypes. In addition, we show that the difference between AJ and C57BL/6J macrophages in controlling *Toxoplasma* growth after stimulation with interferon gamma and tumor necrosis factor alpha mapped to chromosome 3, proximal to the Guanylate binding protein (*Gbp*) locus that is known to modulate the murine macrophage response to *Toxoplasma*. Using an shRNA-knockdown strategy, we show that the transcript levels of an RNA helicase, *Ddx1*, regulates strain differences in the amount of nitric oxide produced by macrophage after stimulation with interferon gamma and tumor necrosis factor. Our results provide a template for discovering candidate genes that modulate macrophage-mediated complex traits.

## Author Summary

Macrophages provide a first line of defense against invading pathogens and play an important role in the initiation and resolution of immune responses. When in contact with pathogens or immune factors, such as cytokines, macrophages assume activation states that range between pro-inflammatory (classical activation) and anti-inflammatory (alternative activation). Even though it is known that macrophages from different individuals are biased towards one of the various activation states, the genetic factors that define individual differences in macrophage activation are not fully understood. Additionally, although macrophages are important in infectious disease pathogenesis, how individual differences in macrophage activation contribute to individual differences in susceptibility to infectious disease is just emerging. We used macrophages from genetically segregating mice to show that discrete transcriptional programs, which are modulated by specific genomic regions, modulate differences in macrophage activation. Murine macrophages differences in controlling *Toxoplasma* growth mapped to chromosome 3, proximal to the Guanylate binding protein (*Gbp*) locus that is known to modulate the murine macrophage response to *Toxoplasma*. Using a shRNA-mediated knockdown approach, we show that the DEAD box polypeptide 1 (*Ddx1*) modulates nitric oxide production in macrophages stimulated with interferon gamma and tumor necrosis factor. These findings are a step towards the identification of genes that regulate macrophage phenotypes and disease outcome.

## Introduction

At the cellular level, innate immune cells, such as macrophages, are central to the development and prevention of infectious diseases. On engagement of surface signaling receptors or pattern recognition receptors (PRRs) such as toll-like receptors (TLRs), RIG-I-like receptors (RLRs) and the cytosolic NOD-like receptors (NLRs), by immune factors such as cytokines or conserved microbial products, macrophages can assume different activation states. The most extreme of these states are the classical (M1, M(IFNG)) and the alternative (M2, M(IL-4)) states, separated by several intermediate activation states [1–3] (We are following the recently described macrophage activation phenotype nomenclature [1]). Ultimately, macrophage activation results in pathogen clearance by downstream antimicrobial effector mechanisms, such as inflammasome activation, or activation of adaptive immune responses [4–6]. Although the outcome of macrophage activation is dependent on the stimulus engaged by the PRRs, emerging empirical data, from both human and mouse studies, indicate that the macrophage genetic background also plays a significant role.

The initiation of immune responses by macrophages can occur in the presence of pro-inflammatory cytokines such as interferon gamma (IFNG), while anti-inflammatory cytokines such as interleukin (IL)-4, and IL-13, prime macrophages for the resolution of immune responses and tissue repair [7–9]. This macrophage ability to initiate and resolve immune responses, while important in regulating immunopathology, can be exploited by pathogens to evade macrophage-associated immunity [10]. Indeed, to disseminate in their hosts most pathogens circumvent macrophage-mediated microbicidal mechanisms by modulating macrophage signaling pathways and activation phenotypes [11–16]. In addition to destroying pathogens, activated macrophages are important mediators in several inflammatory pathologies, including atherosclerosis, diabetes and cancer [17]. Studies in mice have linked several macrophage- or immune-related genes, such as *Nramp1/Slc11a1*, *Icsbp1*/*Irf8*, *Csfgm*, and *Nos2*, with the development of several infectious diseases, including salmonellosis, toxoplasmosis, and leishmaniasis [18–20]. Although the compendium of macrophage- or immune-related genes that modulate infectious disease pathogenesis is broad, the role of individual differences in macrophage activation phenotypes in determining individual differences in susceptibility to infectious disease is just emerging [21–25]. Furthermore, the genetic basis for individual differences in macrophage activation phenotypes has not been identified. Macrophage activation is likely modulated by complex gene and metabolite networks that cannot be defined one gene at a time, thus the difficulty in unraveling the genetics of macrophage activation. This hypothesis is reinforced by results from genetic perturbation experiments that have revealed multiple genes that individually modulate macrophage activation phenotypes, including *IRF8*, *PPARG* and *AKT2* [26, 27]. Empirical data show that macrophages display distinct transcriptional programs in response to infectious and inflammatory stimuli [22] and that this macrophage response differs between genetically segregating individuals [22–25]. Our hypothesis is that inter-individual differences in susceptibility to infectious disease are partly due to genetic differences in the macrophage response to pathogens.

Quantitative trait locus (QTL) analyses have been used to elucidate the complex genetic basis for many traits in humans and model organisms [28, 29]. However, the region spanned by individual QTL is often large and encompasses multiple genes, making the transition from QTL to individual genes influencing disease (quantitative trait gene, QTG) difficult. It has been shown that differences in the abundance of certain transcripts can explain phenotypic variations between individuals [30, 31]. Forward genetics approaches that combine traditional QTL mapping with expression quantitative trait mapping (eQTL; in which case transcript abundance is the quantitative trait) [32] are increasingly being used to successfully transition from QTL to QTG [33–35]. Traditional QTL analysis will identify the genomic regions affecting trait variation, while eQTL analysis can help in understanding which genes, pathways, and biological processes are also under the influence of a given QTL. By examining the relationship between transcript location, the location of the eQTL and the pleiotropic effects of the eQTL, tools of systems biology such as network and functional analysis can be used to further delineate the complex genetic interactions modulating complex traits and reconstruct genetic pathways that underlie such traits [33–35].

In this study, we used the AXB/BXA recombinant inbred (RI) mice to investigate the relationship between the macrophage genotype and their response to inflammatory stimuli or infection with *Toxoplasma*, an obligate intracellular protozoan parasite. The AXB/BXA mice are derived from an initial reciprocal cross of AJ (A) and C57BL/6J (B) mice followed by multiple rounds (≥20) of inbreeding resulting in a stable mosaic of blocks of the parental alleles in their genomes [36]. These mice have been used to investigate the development and susceptibility to a variety of infectious and inflammatory diseases [19, 37–40]. Importantly, the parental strains, AJ and C57BL/6J, differ at key loci that regulate immune responses, including the complement 5 a (*C5a*) [41] and interleukin 3 receptor alpha (*Il3ra*) [25, 42]. These mice also exhibit differences in the amount of IL-10 and tumor necrosis factor (TNF) produced in response to bacterial infection [43, 44]. Furthermore, the parental AJ and C57BL/6J vary in susceptibility to a variety of pathogens, including *Staphylococcus aureus*, *Toxoplasma gondii* and *Trypanosoma cruzi* [38, 44–47]. By linking QTL analyses of defined macrophage phenotypes and macrophage transcriptional profiles, captured by high-throughput RNA-sequencing, we have identified many loci that affect the macrophage response to inflammatory stimuli and infection. These loci could provide the foundations for further studies in identifying the genetic basis for the differences in susceptibility to inflammation and infection in these mice. As an example, we report that differences in nitric oxide (NO) production, in AJ and C57BL/6J macrophages is due to differences in the expression of the RNA helicase *Ddx1*.

## Results

### AJ and C57BL/6J BMDM differ in their response to infectious and inflammatory stimuli

**Fig 1:**
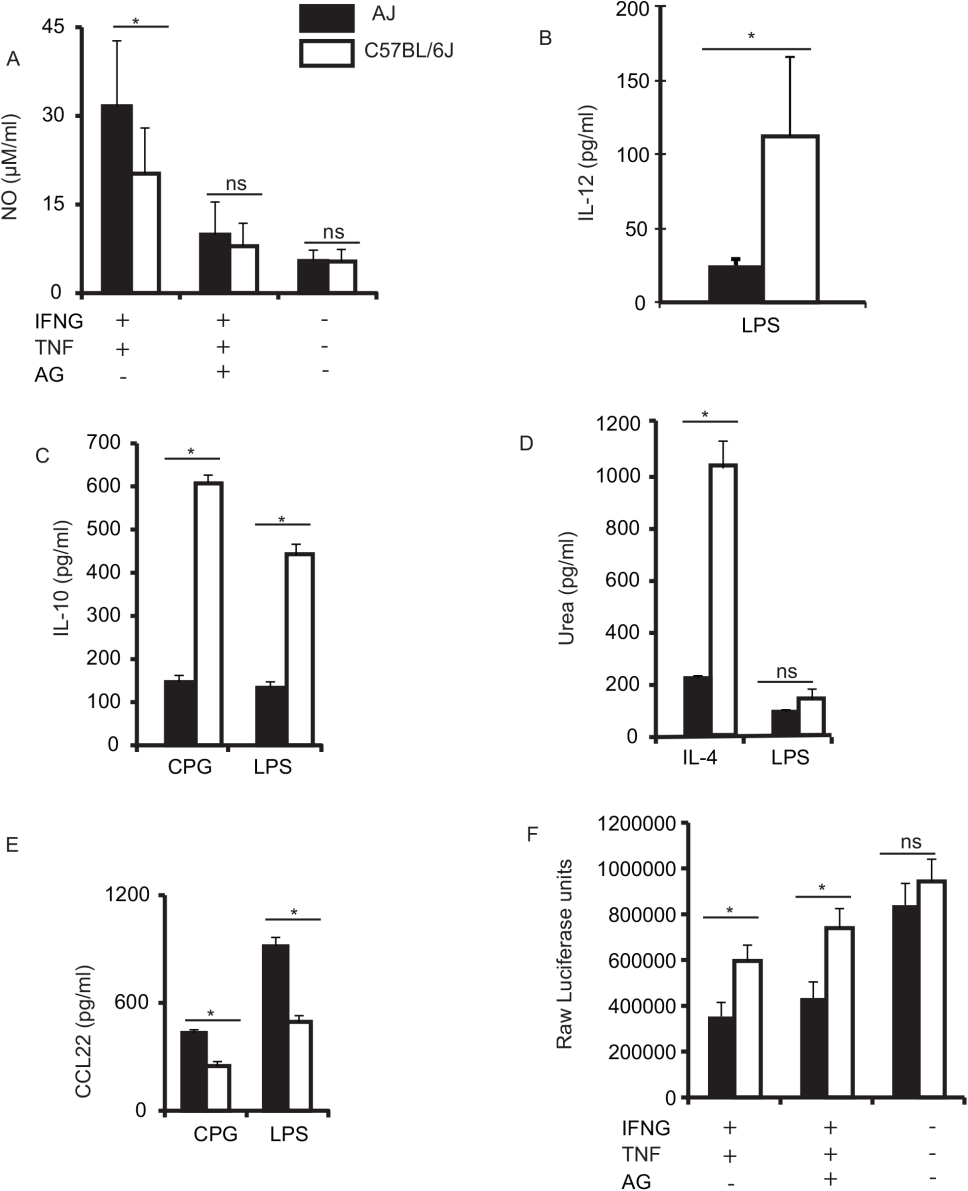
BMDM from AJ and B6 mice differ in their response to various stimuli. A) Stimulation of macrophages for ~ 18 hrs with IFNG+TNF resulted in high production of NO by AJ macrophages (black bars), while B) stimulation with LPS led to higher production of IL-12 in C57BL/6J (B6) macrophages (white bars). C) Stimulation of murine macrophages with LPS or CPG resulted in significantly higher production of IL-10 in B6 macrophages compared to the AJ macrophages. D) B6 macrophages stimulated with IL-4 produced significantly more urea, compared to AJ macrophages, while LPS stimulation induced low amounts of urea in both AJ and B6 BMDM. E) AJ macrophages stimulated with CPG or LPS produced significantly higher amounts of the chemokine CCL22 compared to B6 macrophages. F) The IFNG+TNF stimulated macrophages have increased toxoplasmacidal activity, which is slightly reduced in the presence of aminoguanidine (AG), an inducible nitric oxide synthase inhibitor. Where there was no detectable amount of cytokine/chemokine measured, including in all control (non-stimulated) BMDM, we do not include cytokine/chemokine data in the figures. Three independent replicates; Mean (SD) * *p < 0*.*05* (Student’s t-test).

Although correlations between genetic variations in immune-related genes and the response to infectious and inflammatory stimuli have been reported in AJ and C57BL/6J (B6) [19, 43, 44], the role of specific immune cells in these phenotypic differences are mostly equivocal. Therefore, we stimulated AJ and B6 bone marrow-derived macrophages (BMDM) with interferon gamma (IFNG) and tumor necrosis alpha (TNF) (IFNG+TNF) or interleukin 4 (IL4). IFNG+TNF induces the classical (M(IFNG)) while IL-4 induces the alternative (M(IL-4)) macrophage activation phenotypes. Additionally, to mimic activation by bacteria and pathogen-associated molecular patterns (PAMPs), we stimulated the macrophages with lipopolysaccharide (LPS) (a component of gram-negative bacteria), or CpG (a synthetic oligonucleotide), respectively. A summary of the stimulation regimen and the corresponding phenotypes measured is shown in S1 Table. Next, we captured the macrophage response to the individual stimulus by measuring M(IFNG) and M(IL-4) markers. For M(IFNG) markers, we measured the amount of nitric oxide (NO) and IL-12, while for M(IL-4) markers, we quantified the amount of urea (a by-product of Arginase I enzyme activity), IL-10 and chemokine (C-C motif) ligand 22 (CCL22) (Fig 1). Finally, because: 1) IFNG is indispensable in the resistance to *Toxoplasma gondii* [48], 2) *Toxoplasma* is a master regulator of macrophage signaling pathways [14], and 3) AJ and B6 mice segregate for susceptibility to *Toxoplasma* [38, 49], we infected non-stimulated or IFNG+TNF-stimulated BMDM with a strain of *Toxoplasma* engineered to express firefly luciferase [50] and assessed parasite growth by measuring luciferase activity, which is often used to approximate *Toxoplasma* burden in *in vitro* or *in vivo* infection models [49–52]. We observed high amounts of NO and CCL22 in AJ BMDM, while the B6 BMDM produced higher amounts of IL-12, IL-10 and urea (Fig 1A–1E). Despite producing high amounts of M(IL-4) markers (urea and IL-10), the B6 BMDM also produced high amounts of an M(IFNG) marker (IL-12) relative to AJ BMDM. Similarly, the high amount of NO, an M(IFNG) marker, produced by AJ BMDM was accompanied by high amounts of CCL22, an M(IL-4) marker. This dual expression of M(IFNG) and M(IL-4) markers is perhaps indicative of complex molecular modulation of macrophage activation or the heterogeneity of macrophage activation phenotypes. Consistent with the known resistance of AJ mice to *Toxoplasma* relative to B6 mice [38, 49], and the divergent BMDM activation phenotypes, *Toxoplasma* growth in the IFNG+TNF-stimulated AJ BMDM was significantly reduced compared to its growth in B6 BMDM (Fig 1F). Thus, the variable susceptibility to *Toxoplasma* in AJ versus B6 observed *in vivo* [38, 49] can be recapitulated *in vitro* using AJ and B6 BMDM. As such, the AJ and B6 BMDM can be used to gain insight into the molecular mechanisms that underlie the differences in AJ versus B6 susceptibility to *Toxoplasma*. Together, the difference in macrophage response to IFNG+TNF stimulation, as evidenced by NO (a key toxostatic effector [53, 54]), and the differences in parasite growth in IFNG+TNF-stimulated BMDM, posit that the variable susceptibility to *Toxoplasma* between AJ and B6 mice is due to innate differences in the macrophage response to the parasite and/or IFNG+TNF.

### The genetic basis for differential macrophage response to infectious and inflammatory stimuli

The differences in response to infectious and inflammatory stimuli in AJ and B6 mice have a genetic component [19, 38, 43]. Therefore, having established differences in parasite growth and activation phenotypes in AJ and B6 BMDM, we sought to establish the genetic basis for the differences in macrophage activation and toxoplasmacidal activity between AJ and B6 using the AXB/BXA mice. We stimulated BMDM obtained from 26 age-matched female AXB/BXA RI mice with IFNG+TNF, IL-4, CpG, or LPS and measured the amount of NO, urea, IL-12, IL-10, and CCL22 produced. We also infected IFNG+TNF stimulated BMDM with *Toxoplasma* that express luciferase and measured relative parasite growth by measuring luciferase activity. These phenotypes exhibited a continuous distribution in the RI mice, which is characteristic of quantitative traits (S1A–S1F Fig).

Due to the stable and unique combination of blocks of parental alleles in their genomes, the AXB/BXA RI mice are particularly suited for quantitative trait locus (QTL) mapping [55]. Therefore, we used the AXB/BXA genetic map (containing 934 informative genetic markers [56]) in a genome-wide scan in R/qtl [57] to identify the genomic regions that modulate differences in AJ and B6 BMDM phenotypes. To correct for multiple testing on the 934 genetic markers, we performed 1000 permutation tests on the individuals relative to their phenotypes [58] to obtain adjusted *p*-values for each QTL (Table 1). Parasite growth in the IFNG+TNF-stimulated BMDM mapped to chromosome 3 (147.7 Mb), proximal to the Guanylate binding protein (*Gbp*) locus (142.6 Mb) that is known to modulate the murine macrophage response to *Toxoplasma* [59–62]. Even though AJ and B6 BMDM display distinct polarization states following IFNG+TNF or IL-4 stimulation, except for IL-12 and CCL22, we did not observe statistically significant QTL peaks for any of the activation phenotypes. Instead we observed a suggestive QTL for NO on chromosome 12 (Table 1) and a second QTL for NO on chromosome 4 (S2A Fig). The additive QTL for NO also mapped to chromosome 4 (S2B Fig). Similar to observations in the parental BMDM, the AJ allele at the chromosome 12 QTL was associated with high amounts of NO (S2C Fig). However, the AJ allele at the chromosome 4 QTL was associated with low amounts of NO (S2D Fig). Although mapping in *cis* to *Arg1* on chromosome 10, the QTL for the amount of urea did not reach statistical significance. Nevertheless, the allele effect at the suggestive urea QTL was consistent with the parental allele effect on urea.

**Table 1:** Linkage analysis of AJ and C57BL/6J phenotypic differences using the recombinant inbred AXB/BXA mice.

Stimulation	Phenotype	QTL position (Chr)	QTL peak position (Mb)	LOD	Adjusted *p* value	Gene location
LPS	CCL22	8	109.50	6.6	*0*.*001*	*Ccl22*, 8 (94.94–94.75)
LPS	IL-12	11	37.48	5.5	*0*.*006*	*IL12p40*, 11 (44.40–44.41)
IFNG+TNF	Nitric Oxide	12	9.21	2.9	*0*.*06* *	*Nos2*, 11 (78.92–78.96)
IL-4	Arginase activity	10	21.12	3.8	*0*.*08* *	*Arg1*, 10 (24.91–24.92)
LPS	IL-10	1	76.52	3.3	*0*.*06* *	*Il10*, 1(131.0–131.0)
IFNG+TNF	Parasite growth	3	147.75	4.1	*0*.*08* *	-
LPS	Arginase activity	1	22.1	2.1	*0*.*77*	*Arg1*, 10 (24.91–24.92)
CPG	IL-10	1	40.6	2.9	*0*.*16*	*Il10*, 1(131.0–131.0)
CPG	CCL22	X	99.93		*0*.*20*	*Ccl22*, 8 (94.94–94.75)
Control	Parasite growth	3	147.75	2.7	*0*.*33*	*-*
IFNG+TNF+AG	Parasite growth	3	147.75	3.4	*0*.*10*	*-*
IFNG+TNF+AG	NO	15	87.67	2.5	*0*.*46*	*Nos2*, 11 (78.92–78.96)

QTL were deemed significant at an adjusted *p< 0*.*01* calculated after 1000 permutations in R/qtl. Shown are the individual phenotypes and the corresponding adjusted *p*-values for each LOD score.

* = Suggestive QTL. Gene location indicates the physical location, chromosome followed by position (Mb) in parenthesis, of the gene coding for the measured phenotype.

Finally, we selected the top 2 QTL for each phenotype (where there was marginal difference between the LOD scores for the second and third largest QTL, we picked both) and grouped the BMDM based on the genotypes at each QTL. We then estimated the QTL inheritance pattern by comparing the values for the corresponding phenotype amongst the genotypes using a one-way ANOVA with Tukey’s-Post-test (StatPlus, AnalystSoft Inc) [63] (Table 2).

**Table 2:** QTL inheritance and allele effects on different traits in AXB/BXA BMDM.

Chr	Stimulation	Trait	Marker	AA (Mean)	BB (Mean)	*P*-value
6	LPS	CCL22	rs3695724	409.2	786.8	*1*.*8e-3*
8	LPS	CCL22	rs6182338	874.8	334.0	*8*.*8e-8*
18	LPS	CCL22	rs3720827	806.7	450.5	*3*.*5e-3*
11	LPS	IL-12	rs13480972	56.7	199.8	*1*.*1e-6*
16	LPS	IL-12	rs4188825	172	69.1	*2*.*3e-3*
17	LPS	IL-12	rs3684732	48.7	145.2	*1*.*9e-3*
4	IFN+TNF	Nitric Oxide	rs3723703	9.9	15.1	*1*.*3e-3*
12	IFN+TNF	Nitric Oxide	rs6176675	14.7	9.2	*4*.*6e-4*
1	IL-4	Arginase activity	mCV23695025	2.5	2.1	*9*.*4e-4*
10	IL-4	Arginase activity	CEL-10_2111704	1.5	1.2	*1*.*1e-1*
1	LPS	IL-10	mCV24115911	204.8	89.9	*3*.*0e-4*
4	LPS	IL-10	gnf04.019.134	114.4	180.3	*4*.*2e-1*
2	IFN+TNF	Parasite growth	D2Mit5	2.4 x 10^5^	2.7 x 10^5^	*1*.*9e-1*
3	IFN+TNF	Parasite growth	gnf03.151.552	2.2 x 10^5^	3.1 x 10^5^	*2*.*8e-5*
1	LPS	Arginase activity	mCV23695025	2.0	1.6	*3*.*2e-3*
5	LPS	Arginase activity	rs13478413	1.8	2.1	*1*.*4e-2*
3	Control	Parasite growth	gnf03.151.552	8.9 x 10^5^	1.2 x 10^6^	*1*.*4e-3*
13	Control	Parasite growth	rs13481799	1.0 x 10^6^	8.1 x 10^5^	*7*.*1e-3*
3	IFN+TNF+AG	Parasite growth	gnf03.151.552	1.9 x 10^5^	3.0 x 10^5^	*6*.*1e-3*
X	IFN+TNF+AG	Parasite growth	CEL-X_66015326	2.7 x 10^5^	2.0 x 10^5^	*8*.*2e-3*
1	CPG	IL-10	rs3677272	3.0 x 10^3^	2.0 x 10^3^	*1*.*2e-3*
10	CPG	IL-10	rs6197175	3.3 x 10^3^	2.2 x 10^3^	*1*.*0e-3*
X	CPG	CCL22	CEL-X_94143306	253.6	96.3	*4*.*7e-3*
2	CPG	CCL22	rs3726974	270.6	106.2	*5*.*3e-3*

AA = Homozygous AJ allele; BB = Homozygous B6 allele. BMDMs were grouped based on their genotypes at the marker on the QTL peak. *P*-values were corrected for multiple testing (26 individuals) using the Bonferroni test.

### Divergent genomic loci modulate macrophage transcriptional response to infectious and inflammatory stimuli

Discrete transcriptional programs modulate the response of immune cells, including macrophages, to stimuli such as pathogens and immune factors [24, 31, 33]. As such transcriptional profiles can be used to gain insight into the intricate and incipient molecular networks that modulate complex traits, such as macrophage activation [24, 34, 64, 65]. Consequently, we investigated whether specific transcriptional programs modulate the differential activation of AJ and B6 BMDM. To do this, we performed high throughput RNA-sequencing (RNA-seq) on BMDM obtained from the same 26 age- and sex-matched AXB/BXA mice described above and their progenitors (28 samples in total), before (resting, controls) and after infection with *Toxoplasma* or stimulation with IFNG+TNF, or CpG. For each sample we generated at least 100 million paired-end reads, except for the IFNG+TNF-stimulated BMDM samples that were sequenced on a single end. Although all the samples were sequenced once, due to the unique recombination of the parental alleles and homozygosity at each of the informative genetic markers (934), there are at least 4 replicates (the minimum number of mice having the same allele at each marker) for each marker. Thereafter, we processed the RNA-seq data as previously described [25]. Briefly, we aligned the RNA-seq reads to the mouse reference genome (NCBI build GRcm38, downloaded from Illumina iGenomes; https://support.illumina.com/sequencing/sequencing_software/igenome.html) using TopHat [66]. To avoid read alignment bias due to sequence polymorphisms between AJ and B6 genomes, we made a synthetic reference genome in which all the polymorphic nucleotides between AJ and B6 were converted to a neutral nucleotide [67]. However, and consistent with a recent report [68], allele bias did not significantly affect read alignment to the genome. On average, about 70% of reads in each sample uniquely mapped to the synthetic genome, which was about 1% less than the number of reads uniquely aligned to the iGenome. Henceforth, unless otherwise stated, all RNA-seq data presented herein were processed using the synthetic genome. Transcript abundance was estimated using Cufflinks [69] and reported as fragment per kilobase exon per million reads (FPKM).

**Fig 2:**
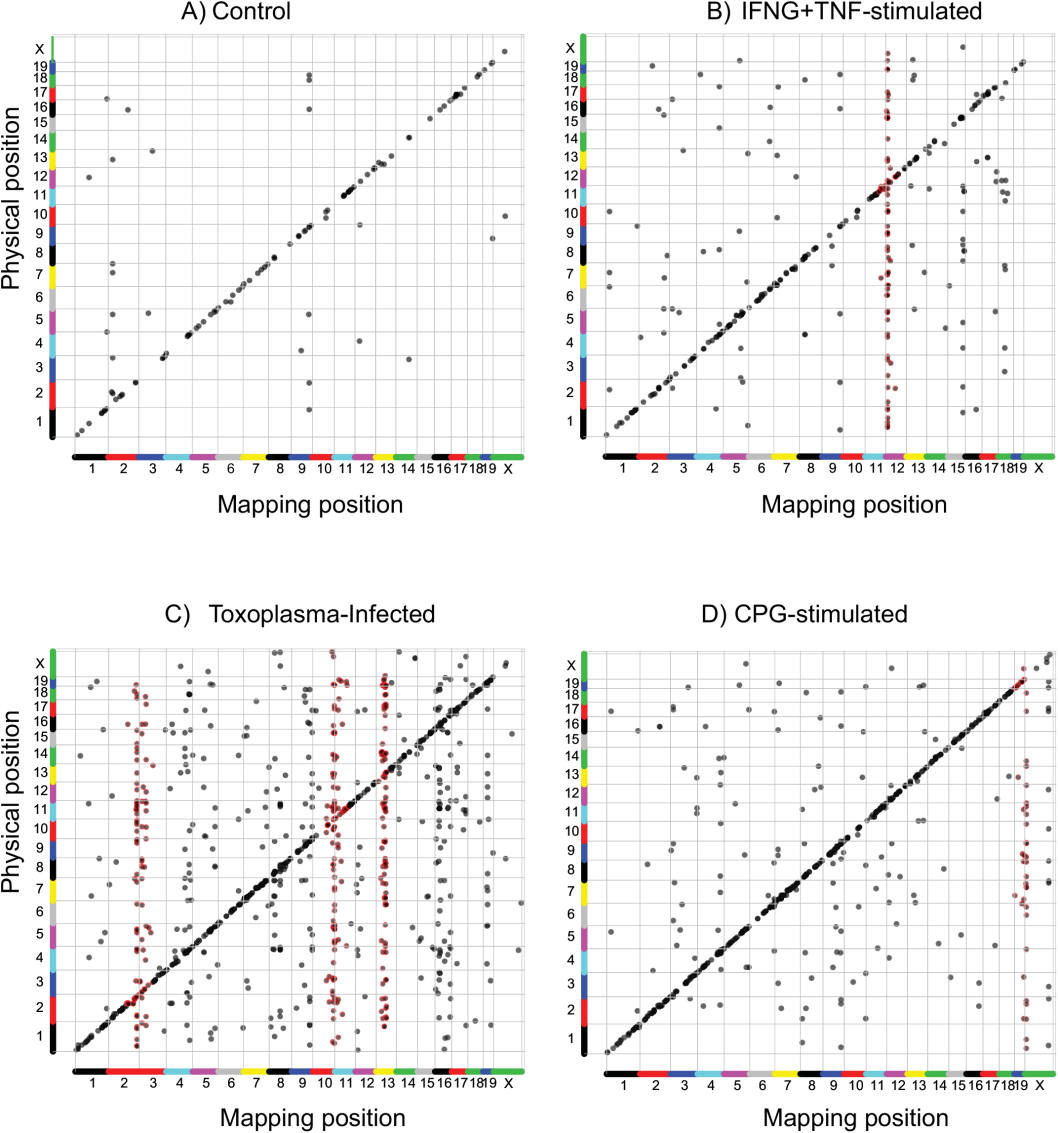
The transcriptional response in BMDM is regulated by stimulation-specific *trans*-loci. Expression quantitative trait loci (eQTL) in the RI mice were mapped in A) non-stimulated, B) IFNG+TNF-stimulated, C) *Toxoplasma*-infected, and D) CpG-Stimulated BMDM. Each dot represents a single eQTL (transcript). Significant eQTL located ≤ 10 Mb from the start of the physical location of the corresponding gene were designated as *cis* mapping (diagonal lines). All other eQTL were designated as *trans*-mapping (vertical lines). eQTL significance was calculated after 1000 permutations and reported at genome-wide thresholds corresponding to FDR ≤ 10%. Red spots identify genes mapping to a *trans*-eQTL hotpot (*trans*-band).

Next, using the FPKM values from each of the 26 RI BMDM and the corresponding AXB/BXA genetic map, we mapped the genomic loci that modulate gene expression (expression QTL, eQTL) [32] using R/qtl [57]. As previously described [25], we performed 1000 permutations to correct for multiple testing across the 934 informative genetic markers in the AXB/BXA cross. Next, we used a false discovery rate (FDR) ≤ 10%, calculated in the qvalue package [70], to correct for multiple testing on the transcripts and to nominate significant eQTL. Finally, to allow for meaningful comparisons, we only included in the downstream analyses, for each stimulation condition, eQTL for transcripts with an average FPKM ≥ 5 across the 26 RI BMDM. The linkage analyses and the subsequent filtering steps were performed separately in resting, IFNG+TNF-stimulated, CpG-stimulated, and *Toxoplasma*-infected BMDM and identified, 131, 367, 688 and 1008 significant eQTL, respectively, dispersed throughout the genome (Fig 2A and 2B and S1A–S1D Dataset). Thus, consistent with previous studies [24, 71], the genetic background influences macrophage gene expression profile. Importantly the different stimuli induced transcriptional programs that were modulated by distinct eQTL hotspots, which can be exploited to unravel the complex molecular networks that regulate macrophage activation states.

### QTL hotspots are enriched for biological processes and putative regulators that modulate the expression of multiple genes

Variable gene expression can be due to sequence or structural variations close to (*cis*) or further removed from (*trans*) the gene itself, such as polymorphism in the promoter regions or at a distal transcription factor, respectively. Consequently, relative to the physical location of the corresponding gene, an eQTL can be categorized as either *cis* or *trans*. Thus, we designated eQTL that co-localized within a 10 Mb genomic window with the corresponding gene as *cis* and all other eQTL as *trans* [72]. Except for the *Toxoplasma*-infected BMDM (407 *cis* vs. 601 *trans*), most of the eQTL were located in *cis* in: resting (99 *cis* vs. 32 *trans*), IFNG+TNF-stimulated (194 *cis* vs. 173 *trans*), and CpG-stimulated (482 *cis* vs. 206 *trans*) BMDM (S1A–S1D Dataset).

Suppose a common locus was to modulate the expression of multiple genes in *trans*, then in linkage analysis, we should expect the eQTL for these genes to co-localize in the vicinity of the common locus forming a *trans*-eQTL hotspot (*trans*-band). Indeed, similar to previous studies [23, 24, 73], and indicative of a common variant regulating the expression of multiple genes in *trans*, we detected *trans*-eQTL hotspots, within a 10 Mb window, in all the samples (Fig 2A and 2B and S1A–S1D Dataset). Because it is possible for eQTL to co-localize by chance alone, we used Bonferroni-corrected *p*-values and Poisson distribution to compute the number of *trans*-eQTL that can co-localize in a 10 Mb genomic window by chance. Using these cutoffs, we identified 2, 3, 5, and 15 *trans*-eQTL hotspots in the resting, IFNG+TNF-stimulated, CpG-stimulated, and *Toxoplasma*-infected BMDM, respectively (S1A–S1D Dataset).

Previously, it was reported that eQTL that localize close to the physical location of the relevant gene (*cis-*eQTL) are a consequence of single nucleotide polymorphisms (SNPs) [72] or larger structural variants (SV), such as insertions and deletions. Thus, we investigated the nucleotide sequence in a 2000 bp window upstream and downstream of the transcription start site (TSS) of all significant eQTL. Consistent with these reports, we found that most *cis*-eQTL were associated with genes reported to have structural or sequence variations within 2000 bp upstream or downstream of their TSS [74, 75]. That is, in control BMDM we found 89 out of 99 (Hypergeometric test *P≤2*.*3e-7*); in the IFNG+TNF-stimulated BMDM we found 156 out of 196 (Hypergeometric test *P≤3*.*3e-10*); in the CpG-stimulated BMDM we found 422 out of 482 (Hypergeometric test *P≤3*.*8e-23*); and in the *Toxoplasma*-infected BMDM we found 369 out of 407 (Hypergeometric test *P≤1*.*6e-*49) *cis*-eQTL with polymorphisms within 2000bp upstream or downstream of TSS. These included genes with known immunological functions such as *Gbp1*, *Gbp2*, *Irak4* and *Srebf1*.

As indicated above, *trans*-eQTL hotspots can be due to transcriptional regulation of several genes by a common genetic variant [76], such as a polymorphic or differentially expressed transcription factor, enhancer or repressor. Alternatively, a *trans*-band can be a result of a differentially expressed or polymorphic signaling protein, such as a cell surface receptor, that could lead to differential activation of transcription factors and the genes downstream of these transcription factors. Consequently, the eQTL localizing at the *trans*-eQTL hotspot are likely to be enriched for binding sites for a common transcription factor(s) physically located at the *trans*-eQTL-hotspot, biological function, or signaling pathway. Therefore, we used gene ontology (GO) [77] and rVISTA [78] to functionally characterize and search for transcription factor binding site enrichment in each of the eQTL in the different *trans*-hotspots (Table 3). We found that the three largest *trans*-bands in the *Toxoplasma* infected BMDM mapped to loci containing genes with either known or putative roles in macrophage inflammatory or metabolic processes, which are important host responses against intracellular pathogens. For instance the *trans*-band on chromosome 13 (117.1–127.8 Mb) overlapped docking protein 3 (*Dok3*), chemokine (C-X-C motif) ligand 14 (*Cxcl14*), and AU RNA binding protein/enoyl-coenzyme A hydratase (*Auh*), which are known to regulate various aspects of cellular inflammatory and metabolic processes [79–81]. Indeed, this *trans*-band was enriched for, among others, “natural killer cell mediated immunity”. Similarly, the *trans*-band in the IFNG+TNF-stimulated BMDM on chromosome 15 (89.6–96.6 Mb), which was enriched for “regulation of leukocyte mediated immunity”, overlapped the interleukin-1 receptor-associated kinase 4 (*Irak4*), known to regulate the immune response to a variety of infectious and inflammatory stimuli [18, 82, 83]. Furthermore, *Irak4* contains genetic insertions and deletions in AJ relative to the reference B6 mouse strain [84]. Thus, the *trans*-bands are functionally enriched in biologically relevant processes and can potentially reveal novel regulators and insight in the complex gene interactions that modulate macrophage response to exogenous stimuli.

**Table 3:** Functional enrichments in the large *trans*-eQTL hotspots in AXB/BXA macrophages following IFNG+TNF-stimulation, CpG-stimulation, or *Toxoplasma*-infection.

Stimulation	*Trans*-band	Functional enrichment	Adjusted *P*-value	Enriched TF	P-value
IFNG+TNF	12 (4.4–13.1)	Purine metabolism	*5*.*72e-05*	*E2f6*	*0*.*0008*
		Nitrogen compound metabolic process	*2*.*95e-05*	*Creb*	*0*.*0001*
CpG	X (139.6–141.1)	Histone H3-K4 acetylation	*1*.*80e-03*	*Sp1*	*4*.*92e-05*
		Positive regulation of nucleic acid-templated transcription	*7*.*91e-03*		
*Toxoplasma*	2 (161.5–168.3)	Negative regulation of histone H3-K27 methylation	*2*.*04e-02*	*Sp1*	*1*.*05e-18*
		Positive regulation of purine nucleotide catabolic process	*5*.*11e-02*	*Egr1*	
				*Zfp148*	*4*.*16e-15*
					*1*.*40e-15*
	10 (117.1–127.8)	Leukocyte differentiation	*2*.*31e-04*	*Zscan10*	*6*.*20e-08*
		Positive regulation of protein deacetylation	*2*.*97e-03*	*Hif1*	
				*Xbp1*	*1*.*94e-05*
					*5*.*37e-04*
	13 (47.0–57.7)	Fc-gamma receptor signaling	*2*.*10e-04*	*Elk3*	*5*.*53e-09*
		Natural Killer cell mediated immunity	*1*.*83e-03*	*Etv5*	
				*Ets2*	*1*.*80e-05*
					*5*.*15e-04*

To identify putative regulators for the *trans*-bands, which can be variable transcription factors or signal transducers, we identified genes that were expressed (average FPKM ≥5), were physically located within 10 Mb on either side of the *trans*-eQTL hotspot, and were differentially expressed or had non-synonymous (NS) polymorphisms in AJ versus B6 mice. As an example, the chromosome 15 *trans*-band (between 79.7–99.7 Mb) contained 81 expressed coding and non-coding genes, 5 of which contained non-synonymous SNPs and 8 exhibited differential expression that mapped in *cis*. Of these genes, we considered *Plxnb2*, *Irak4*, and *Apobec3* to be good candidates since they are known to be involved in immune response pathways [82, 85, 86], similar to the functional enrichment observed for the chromosome 15 *trans*-band. Additionally, these genes are polymorphic in AJ compared to B6. Due to insertions and deletions [87] and its function as an immune signaling adaptor, we considered *Irak4* to be a strong candidate regulator for the chromosome 15 *trans*-band. Hence, we used shRNA to knockdown *Irak4* in the IFNG+TNF-stimulated macrophages. Enrichment analysis on the perturbed genes following *Irak4* knockdown revealed an overrepresentation (*p = 0*.*005*) of several chromosome 15 *trans*-eQTL (Table 4). shRNA-knockdown of the other putative candidate genes identified in this study did not perturbed most of the chromosome 15 *trans*-band eQTL (S1E Dataset), indicative of a specific effect of *Irak4* knockdown on this *trans*-band.

**Table 4:** Genes from the chromosome 15 *trans*-band in IFNG+TNF-stimulated BMDM that were perturbed by at least 2 fold after *Irak4* knockdown and IFNG+TNF stimulation of murine BMDM.

Gene Id	Fold change	Biological process
Pcdh7	2.40	Cell adhesion
Siglec1	2.16	Endocytosis
Aim1	2.46	-
B4galt6	2.64	Sphingolipid biosynthetic process
Slc37a2	2.18	Glycerol-3-phosphate metabolic process
Rgl1	3.22	Small GTPase mediated signal transduction
Slc16a10	2.25	Transmembrane transport
Cd72	3.34	
Cd300lb	2.66	
5430435G22Rik	2.50	Negative regulation of toll-like receptor 4 signaling pathway
Tlr9	2.20	Toll-like receptor signaling pathway
Cd24a	2.84	Regulation of cytokine-mediated signaling pathway
Arhgap18	2.28	Signal transduction
Irak4	-2.16	Cytokine-mediated signaling pathway

### Leveraging eQTL analysis to identify candidate genes that modulate macrophage response to exogenous stimuli

Transcriptional networks capture the connectivity between genes modulating complex phenotypes and may provide a means to unravel the molecular mechanisms underlying complex traits. Because *cis* genetic variants account only for the phenotypes related to the gene they modulate, we reasoned that they are not good prototypes to illustrate how transcriptional, linkage and network analyses can be leveraged to systematically elucidate the genetic basis of a complex trait. Therefore, we used *trans* genetic variants, which potentially modulate multiple phenotypes, and followed a step-wise procedure [34] to identify the relationship between transcript levels, QTL, and BMDM phenotypes. First, to gain insight into the transcriptional architecture that modulate macrophage response to stimuli, we constructed gene co-expression network modules for each macrophage stimulation condition using the topological overlap matrix (TOM) in the weighed gene co-expression network analysis (WGCNA) program [88]. Next, using the eigenvalues for each module, we made correlations between each module and macrophage phenotypes. As proof of principle, we used this approach to nominate candidate genes that modulate the amount of NO produced in IFNG+TNF-stimulated BMDM. Because the amount of NO varied in the BMDM after IFNG+TNF, we correlated the co-expression modules with the amount of NO produced in the IFNG+TNF-stimulated BMDM (S3 Fig). Subsequently, we identified 4 modules (identified as white, light yellow, blue, and tan) that showed significant (*P ≤ 0*.*01*) correlation with NO levels (S3 Fig). It is important to note that, apart from being arbitrary identifiers for each module, the color code used to name each module reveals no further information. Each module, however, contains co-expressed genes (i.e. genes that exhibit transcriptional correlation across the 26 IFNG+TNF-stimulated BMDM). Of the 4 modules, the “white” module showed the greatest association with NO, hence we used it to illustrate our approach. Because the module-trait relationship is based on the correlation of the module eigenvalue with the amount of NO, not all the genes in the module will show significant association with the trait (amount of NO produced in the BMDM). Therefore, we further filtered the genes in the “white” module based on their individual relationship with NO (gene significance) (S1F Dataset). Expectedly, the eQTL for most of the genes in the module were localized on chromosome 12 and chromosome 4, the locations for NO QTL. To identify the potential regulator for cellular amounts of NO, we reasoned that if two genetic traits are both modulated by the same genetic variant, then the QTL for the two traits will co-localize at the common genetic variant [34]. Therefore, to further narrow down the significant genes and to identify the transcriptional network that likely modulate genetic differences in cellular amounts of NO produced after IFNG+TNF-stimulation, we searched for genes with eQTL that overlapped with the NO QTL on chromosome 12 (4.4–13.1 Mb) and found 11 eQTL (S1F Dataset). These eQTL were functionally enriched for “oxidoreductase activity” (*p* = *8*.*322e-04*) and “nitrogen compound metabolism” (*p = 1*.*345e-03*) in gene ontology.

**Fig 3:**
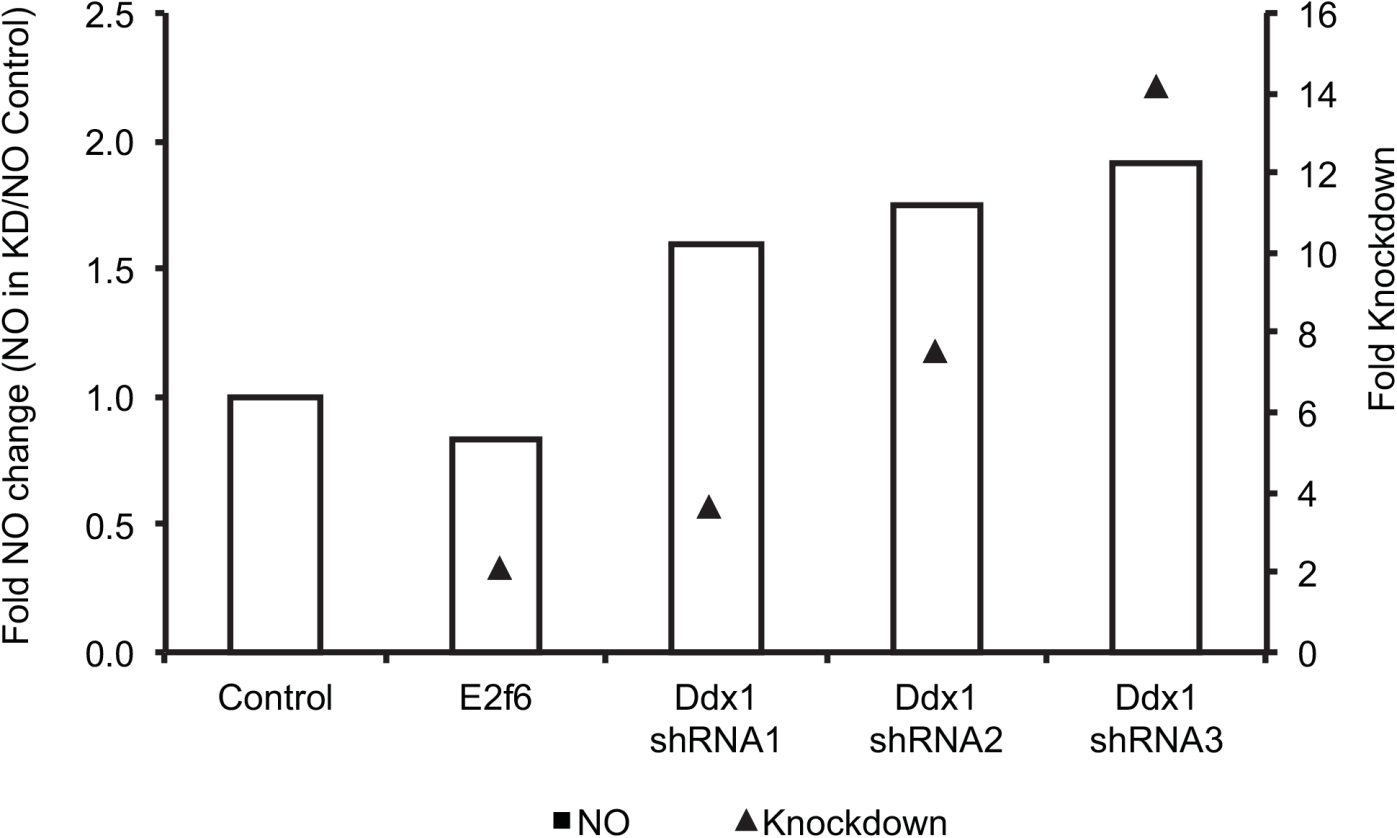
shRNA- mediated *Ddx1* knockdown in C57BL/B6J immortalized macrophages relieves NO inhibition. Fold knockdown of *Ddx1* and fold change in NO levels are relative to the *Ddx*1 expression and the amount of NO in cells transduced with control shRNA (*LacZ*), respectively. Knocking down *E2f6*, the other candidate gene at this locus, did not affect the amount of NO produced. Shown are values of NO (μM) fold change obtained from two independent experiments using three different shRNA constructs. The knockdown level is indicated by the black triangles. Fold knockdown was calculated using the 2^deltadelta^ *Ct* method. The shRNA transductions and NO measurements were done in 3 independent replicates.

Because the common regulator for both NO levels and the chromosome 12 *trans*-band can be a polymorphic or differentially expressed transcription factor or signaling receptor physically located at the chromosome 12 *trans*-band locus, we narrowed the putative regulators by searching for *cis*-eQTL or expressed polymorphic genes at the chromosome 12 locus. *Ddx1*, a transcriptional regulator of cell cycle, maps in *cis* at this locus, and was considered a putative candidate for NO. Additionally, we used the Transcriptional Regulation Inference from Genetics of Gene Expression (Trigger) program [89] to establish the causal relationship between NO and the genes on chromosome 12, including *Ddx1*. Trigger utilizes randomized genetic backgrounds and phenotypes to test for causality between phenotypes that are linked to the same locus e.g. *Ddx1* and NO. Of the chromosome 12 genes, only *Ddx1* exhibited a minimum *p*-value (≤0.05) for causal relationship. As expected, the reciprocal analysis did not show NO or any of the chromosome 12 *trans*-eQTL as causal for *Ddx1* differential expression. Indeed, shRNA-mediated knockdown of *Ddx1* in immortalized B6 macrophages resulted in an increase in the amount of NO produced after IFNG+TNF-stimulation (Fig 3). Similar to NO, *Ddx1* transcript abundance in IFNG+TNF-stimulated macrophages mapped to two loci on chromosome 4 and 12. However, *Ddx1* transcript abundance exhibited an inverse relationship with the amount of macrophage NO at both loci (S4A–S4E Fig). Thus, we concluded that the expression of *Ddx1*, which is higher in B6 compared to AJ BMDM, inhibited NO production in the B6 macrophages.

Due to the important role macrophages play in the pathology of various intracellular pathogens [90], we investigated whether there were overlaps between our *trans*-eQTL hotspots and other disease QTL in the AXB/BXA RI mice available in webQTL [91] and found several (S1A–S1D Dataset). For example the QTL for *Listeria monocytogenes* proliferation, which is attributed to macrophage inflammatory response [92], and susceptibility to hepatitis virus [93] overlapped the *trans*-eQTL hotspots on chromosome 15 and 7, respectively, in the IFNG+TNF-stimulated macrophages. Considering that IFNG+TNF is important in the pathogenesis of *Listeria* and *Hepatitis* infections [94–96], it is likely that these *trans*-bands harbor genes that modulate the outcome of infection with these and other pathogens.

## Discussion

The activation of macrophages, in response to stimuli such as microbial components or immune factors, into the broadly defined classical, M(IFNG), and alternative, M(IL-4), phenotypes, determines whether they initiate or resolve immune responses. As gatekeepers against invading pathogens, the macrophage activation phenotype is essential in determining the persistence or resolution of infection. Thus, the hypothesis is that for infection to persist, the pathogen has to “trick” host macrophages to assume the “wrong” activation state and that variation in susceptibility to infection between hosts is due to genetic differences in macrophage response to the pathogen. Indeed empirical evidence shows that many pathogens can alter the macrophage activation to a phenotype that is favorable for its replication and persistence. For example, virulent strains of the intracellular parasite *Toxoplasma gondii*, which is vulnerable to M(IFNG) macrophages, induces the M(IL-4) phenotype in macrophages [12, 14, 97]. Similar observations have been made in leishmaniasis, in which the extent of macrophage modulation is dependent on the host [98]. On the other hand, macrophages from genetically segregating hosts have been shown to exhibit differential activation states, as captured by transcriptional and cytokine profiles [21, 71, 99–101]. Despite these documented pathogen-macrophage interplays and the variable inter-host macrophage activation phenotypes, the genetic basis for individual differences in macrophage polarization is just emerging. We describe differential activation of macrophages from the genetically segregating AJ and C57BL/6J (B6) mouse strains, which we have linked to the variable response to the obligate intracellular parasite, *Toxoplasma gondii*. Our *in vitro* model, which obviates the interference by other immune cells and involves naïve bone-marrow derived macrophages as opposed to elicited peritoneal macrophages, indicates that when stimulated with equal amounts of cytokines, AJ-derived macrophages produce a stronger M(IFNG) phenotype relative to B6 (as measured by NO). Additionally, we used a panel of genetically diverse recombinant inbred (RI) mice, derived from AJ and B6 mice, to investigate the specific loci responsible for this variable macrophage activation and the associated phenotypes.

In mice, nitric oxide (NO) production and L-Arginine metabolism are often used to define the M(IFNG) and M(IL-4) macrophage activation phenotypes, respectively [100, 102]. While the cellular levels of these factors vary between genetically divergent individuals and are known to contribute to the differential response to infection [101, 103, 104], the genetic loci that predispose macrophages to either the M(IFNG) or M(IL-4) phenotypes are not known. The general assumption is that a single locus will determine whether individual macrophages assume the M(IFNG) or M(IL-4) state. Results from the current study contradict this assumption and suggest that macrophage activation, as measured by levels of NO, IL-12, CCL22, IL-10 and Arginase I activity, is modulated by several loci, some with antagonistic effects. While the limited number of mice available from AXB/BXA RI line may partly explain the lack of statistically significant QTL for most of the macrophage phenotypes in this study, we submit that the genetic factors that modulate macrophage polarization form complex interaction networks that are not linked to a single locus. This conclusion is supported by the identification of two loci with contrasting effect on NO levels, the high levels of IL-12 and urea in B6 BMDM, and the high levels of NO and CCL22 in AJ BMDM. Furthermore, our observation of significant QTL peaks for IL-12 and CCL22 portends that the complex regulation of other phenotypes, such as NO, rather than the few number of mice, maybe the reason for the insignificant QTL peaks. Apart from the individual macrophage activation phenotypes, we did not observe the convergence of all the M(IFNG) (IL12, NO) at a single locus and the M(IL-4) phenotypes (IL10, CCL22 and urea) at another locus, instead each phenotype localized to a unique locus, again reinforcing the complex molecular circuits that modulate the M(IFNG) and M(IL-4) macrophage states. Although, the B6 mice carry a dominant negative mutation in the *Slc7a2* gene, which is involved in L-Arginine transport and is postulated to contribute to the differential metabolism of L-Arginine in B6 relative to other mouse strains such as BALB/c [105], the QTL for L-Arginine metabolism did not localize to chromosome 8, which is the physical location of *Slc7a2*. Instead, the QTL for urea (a measure of Arginase activity) mapped to chromosome 10, proximal to *Arg1*, while the QTL for NO mapped to chromosome 12, respectively. It is plausible that both the transport and metabolism of L-Arginine contribute to the difference in urea and NO production in AJ and B6, hence the lack of significant QTL for these traits.

Modulation of host cellular signaling and transcriptional pathways by *Toxoplasma* is known to aid in immune evasion by the parasite and is achieved via the secretion of polymorphic effector proteins localized in the rhoptry and dense granule organelles [37, 106–108]. Specifically, the secretion of the polymorphic dense granule protein (GRA15) by the avirulent type II, and rhoptry kinase (ROP16) by the virulent type I *Toxoplasma* strains, is known to elicit classical and alternative macrophage activation, respectively [12]. Additionally, the secretion of these two *Toxoplasma* effector proteins is known to modulate intestinal pathology in the susceptible B6 mice [37]. However, to the best of our knowledge, the potential role of macrophages in *Toxoplasma*-induced intestinal pathology has not been shown. Furthermore, even though AJ and B6 are known to diverge in their response to *in vivo Toxoplasma* infection phenotypes, there has been no study showing that this variable response is due to a differential response of their macrophages to either IFNG or to the parasite itself. Together, the current study and our previous work [49], provide compelling evidence that macrophages may play an important role in *Toxoplasma* pathology. We postulate that alternative macrophage activation by *Toxoplasma* [12], and the differential AJ and B6 macrophage response to *Toxoplasma* and IFNG+TNF, provide the intersection of host-parasite interaction that harbors candidate genes mediating murine toxoplasmosis. *Toxoplasma* virulence appears to be related to its ability to skew macrophages towards alternative activation, which is abetted in susceptible animals, such as B6 mice.

In conclusion, our findings provide an extensive genetic analysis of the macrophage signaling processes in response to exogenous stimuli. Because activation of macrophages by IFNG and/or TNF confers resistance to a wide range of intracellular pathogens and human diseases, and because susceptibility loci for some of these phenotypes overlap, it is expected that this study will provide a framework to help identify candidate genes that mediate some of these disease phenotypes.

## Material and Methods

### Ethics statement

All animal experiments were performed in strict accordance with National Institutes of Health Guide for the Care and Use of Laboratory Animals and the Animal Welfare Act. The Massachusetts Institute of Technology Committee on Animal Care (assurance number A 3125–01) approved all protocols. All mice were maintained in specific pathogen-free conditions and euthanasia was performed in controlled CO_2_ chamber as approved by the MIT Animal Care Committee.

### Primary bone marrow derived macrophages and parasites

Bone marrow-derived macrophages (BMDM) were obtained from 6–8 weeks old AJ, C57BL/6J and 26 female AXB/BXA recombinant inbred mice (Jackson Laboratories). Marrow cells were obtained from each mouse by flushing the femur and tibia with cold phosphate buffered saline (PBS; GIBCO-Invitrogen). The cells were then centrifuged at 500 x g for 5 minutes at 4°C and re-suspended in 4 ml red cell lysis buffer (Sigma) and incubated on ice for 5 minutes. Next, the cells were passed through a 70 μm cell strainer (BD Biosciences) and centrifuged at 500 x g for 5 minutes at 4 °C. The cells from each mouse were subsequently grown on four 10 cm non-tissue culture petri dishes (Corning) in Dulbecco's modified Eagle's medium (DMEM; GIBCO-Invitrogen) supplemented with 10% heat-inactivated fetal bovine serum (FBS; HyClone), 2 mM L-glutamine, 1 mM sodium pyruvate, 1X MEM nonessential amino acids, and 50 μg/ml each of penicillin and streptomycin. To differentiate the cells into macrophages, the DMEM was conditioned with 20% L929 cell supernatant (containing GM-CSF (40 ng/ml), hereafter 20% L929). After incubating the cells at 37°C and 5% CO_2_ for 3 days, the non-adherent cells were pipetted into 50 ml tubes and centrifuged at 500 x g for 5 minutes at 4°C and seeded in new 10 cm petri dishes. Simultaneously, the old 10 cm petri dishes were topped up with fresh media supplemented with 20% L929 i.e. in the end, for each mouse there were 8 petri dishes. After further incubation at 37°C and 5% CO_2_ for 4 days, the BMDM were harvested and stored in liquid nitrogen (5 million cells/ aliquot). The BMDM yield for each mouse strain ranged between 60–150 million cells, with the B6 mouse consistently producing more cells. This protocol has previously been shown to yield pure (>99%) macrophages [12]. A Pru (type II) *Toxoplasma gondii* strain engineered to express firefly luciferase and GFP (Pru Δ*HXGPRT* A7) [109], maintained in the laboratory by serial passage on Human Foreskin Fibroblasts (HFF), was used for all infections.

### Immortalized macrophages

To immortalize macrophages, we used J2 recombinant retrovirus [110] produced from ψCREJ2 cells (a generous gift from John MacMicking, Yale University School of Medicine). The J2-expressing cells were grown to confluency in DMEM medium supplemented with 10% FBS (D10). The medium containing retroviral particles was collected and passed through 0.45 μM low protein-binding filters (Millipore). In parallel, 5 x 10^6^ primary BMDM from AJ and C57BL/6J, obtained as described above, were thawed and grown for 2 days in DMEM medium supplemented with 10% FBS and 20% L929. After 2 days, the medium was replaced with the filtered J2-retrovirus-containing medium supplemented with 50% L929. After 24 hrs, the media was replaced with fresh D10 medium supplemented with 25% L929. Media was subsequently changed after every 24hrs with concomitant reduction in L929 until 10% L929 concentration was reached. Immortalized cells were harvested and stored in liquid nitrogen until use.

### *In vitro* measurements

Unless otherwise stated, a total of 10^4^ /well immortalized BMDM (iBMDM) or 10^5^ /well primary BMDM were used in all the *in vitro* assays. Unless stated otherwise, before stimulation or infection, the primary BMDM or iBMDM were seeded overnight in D10 supplemented with 20% L929. Cellular nitric oxide levels were measured using the Griess reagent procedure on supernatant from non-stimulated or stimulated cells. Arginase activity was measured by quantifying the amount of urea as previously described [111]. Briefly, L-arginine was added to cell lysates and incubated at 37°C. After 1 hour, 175μl of an acid mixture containing sulfuric acid/phosphoric acid/water (H_2_SO_4_/H_3_PO_4_/H_2_O) in a 1:3:7 ratio, was added to each well to stop the enzymatic reaction. Urea was quantified calorimetrically at 540 nm after adding 1.25 μl of 1-phenyl-1,2-propanedione-2-oxime (ISPF) and heating at 95°C for 30–60 minutes. This procedure abrogates interference from other metabolites generated, such as L-citrulline [111]. IL10, and IL12 were measured on the relevant cell supernatants using ELISA kits as previously described [12]. For parasite growth assay, cells were either left unstimulated (control) or stimulated with recombinant mouse IFNG (100 ng/ml, Peprotech) and TNF (100 ng/ml, AbD serotec) for ~18 hr. The supernatant was removed for nitric oxide assay and replaced with D10 containing *Toxoplasma* at an MOI ~1. The parasites were allowed to infect and replicate for 24 hrs before luciferase activity was measured using a luciferase assay kit (Promega) according to the manufacturer recommendations.

### RNA sequencing

Primary BMDM were plated (3 x10^6^) overnight before stimulation or infection. For the stimulated samples, IFNG (100 ng/ml) and TNF (100 ng/ml) were added to each well for 18 hrs, while for the infected samples, a type II strain of *Toxoplasma* (Pru) was added to the confluent BMDM at an MOI of 1.3 for 8 hrs. Total RNA (Qiagen RNeasy Plus kit) was then isolated from the non-stimulated and non-infected cells (controls plated overnight), stimulated, and infected cells and the integrity, size, and concentration of RNA checked (Agilent 2100 Bioanalyser). The mRNA was then purified by polyA-tail enrichment (Dynabeads mRNA Purification Kit; Invitrogen), fragmented into 200–400 base-pairs, and reverse transcribed into cDNA before Illumina sequencing adapters were added to each end. Libraries were barcoded, multiplexed into 4 samples per sequencing lane in the Illumina HiSeq 2000, and sequenced from both ends resulting in 40 bp reads after discarding the barcodes. Our preliminary RNA-seq experiments with infected BMDM have shown that with 4 samples per lane, we still obtain enough read coverage for reliable gene expression analysis.

### Gene expression measurements

Reads were initially mapped to the mouse genome (mm9) and the *Toxoplasma* (ME49 v8.2) genome using Bowtie (2.0.2) [112] and Tophat (v2.0.4) [66]. We then estimated gene expression levels in cufflinks (v2.0.0) [113] using the Illumina iGenomes refseq genome annotation (NCBI build 37.2) with the multi-read, compatible-hits corrections and upper quantile normalization options enabled. Because the reference genome to which we mapped the RNA-seq reads is based on the C57BL/6J genomic sequence, and due to the known polymorphisms between the AJ and C57BL/6J, we suspected that biases introduced at the read mapping stage might affect our expression results. To mitigate this potential bias towards the reference allele, we created a copy of the mouse genome in which all the known single nucleotide polymorphisms (SNPs) between AJ and B6, as annotated by the Wellcome Trust Sanger Institute sequencing (ftp://ftp-mouse.sanger.ac.uk/current_snps/), were converted to a third (neutral) nucleotide that is different from both the reference and AJ allele [67]. However, this did not substantially change the average proportion of uniquely mapped reads or expression profiles of individual genes in all the samples. In the end we used the mapping data generated from the synthetic genome to quantify gene expression levels.

### Quantitative Trait Locus (QTL) mapping

To map QTL, we used 934 AXB/BXA genetic informative markers obtained from http://www.genenetwork.org. For all the *in vitro* measurements and gene expression linkage analysis, a genome-wide scan was performed using R/qtl [57]. Significance of QTL logarithm-of-odds (LOD) scores was assessed using 1000 permutations of the phenotype data [114] and the corresponding p-values reported. For the cellular phenotypes, QTL significance was reported at a genome-wide threshold corresponding to *p* < 0.05. However, for eQTL mapping, we further corrected for multiple testing on the multiple transcripts by using the *p*-values to estimate false discovery rate (FDR) in the qvalue package [115] and reported significant eQTL at FDR ≤ 10%. To identify *cis-* and *trans*- eQTL, we computed the distance from the position of the eQTL and the start of the physical location of the corresponding gene and designated any eQTL located <10 Mb from the corresponding gene as *cis*, otherwise *trans* eQTL. The procedure used to determine *trans*-bands has previously been described [25].

### Validation of candidate regulators

We used shRNA to probe for functional or regulatory significance of some of the candidates identified in our analysis. To do this, we used C57BL/6J immortalized bone marrow-derived macrophages, described above. One day after plating, we added shRNA constructs containing a puromycin resistance marker (RNAi Platform, Broad Institute) in the presence of 8 μg/ml polybrene, followed by centrifugation at 800 x g for 2 hrs at 37°C. At the end of the spinfection, the cells were incubated for an additional 24 hrs at 37°C in 5% CO_2_. The cells were then grown in fresh cell culture medium for an additional 24 hrs before adding 4 μg/ml puromycin. Transcript knockdown was measured by quantitative reverse transcriptase polymerase chain reaction (qRT-PCR) using the KAPA SYBR FAST Universal 2X qPCR Master Mix (KAPA Biosystems) on a LightCycler qPCR instrument (Roche). Fold knockdown was measured using the 2 delta-delta method [116] relative to LacZ-shRNA transduced cells. The puromycin-selected cells were either left stimulated or stimulated with IFNG+TNF, as described above, and the cell supernatant collected for nitric oxide assay.

### Data access

The microarray data is available at the NCBI Gene Expression Omnibus archive under accession number GSE47046.

## Supporting Information

S1 Table A summary of the phenotypes measured in each stimulation. (DOC)

S1 Fig Macrophage phenotypes in AXB/BXA recombinant inbred mice. A) Parasite growth (raw luciferase readout) in IFNG+TNF-stimulated BMDM, and B) Cellular amounts of NO in IFNG+TNF-stimulated macrophages, C) Amount of Urea (Arginase enzyme activity) in IL-4-stimulated BMDM, D) amount of IL-10 in LPS-stimulated BMDM, E) amount of IL-12 in LPS-stimulated BMDM, and F) amount of CCL22 in CpG-stimulated BMDM, exhibit quantitative trait quality. Three independent replicates; Mean (+SD) * *p < 0*.*05* (Student’s t-test).
(EPS)

S2 Fig Two loci with antagonistic effect modulate the amount of nitric oxide in IFNG+TNF-stimulated macrophages. A) Primary genome-wide scans for the amount of NO produced in BMDM stimulated with IFNG+TNF reveal loci on chromosome 4 and 12. B) Using the marker at the NO major QTL on chromosome 12 as a covariate narrows the QTL peak on chromosome 4 (Red line). The effect of the two QTL on NO is antagonistic with the allele on C) chromosome 4 conferring higher amounts of NO in B6 BMDM, while D) the allele on chromosome 12 is associated with lower amounts of NO in B6 BMDM.
(EPS)

S3 Fig Correlating cellular amounts of nitric oxide (NO) with gene expression modules. Using WGCNA, we constructed the co-expression modules for the genes in the resting (control) and IFNG+TNF-stimulated BMDM. A) The modules were then correlated with the amounts of NO in IFNG+TNF-stimulated BMDM. The correlation value for each module (named as colors) is included in each box and the corresponding *P* value in brackets. The white module showed the greatest correlation with NO in the IFNG+TNF-stimulated BMDM. B) Shows the gene significance (correlation potential) of each gene in the white module with NO.
(EPS)

S4 Fig *Ddx1* and NO QTL co-localize on chromosome 4 and 12. A) The *Ddx1* eQTL peak (black) and NO QTL peak (red) co-localize on chromosome 4 and 12. B) Relative to the B6, the AJ allele at the *Ddx1* eQTL on chromosome 12 is correlated with low expression levels of *Ddx1* (average expression across strains with each of the indicated genotype at this locus), but is C) correlated with high amounts of NO in IFNG+TNF-stimulated BMDMs. D) Conversely, the AJ allele at the *Ddx1* eQTL on chromosome 4 is linked with higher expression of *Ddx1* and E) lower cellular amounts of NO in IFNG+TNF-stimulated BMDMs
(EPS)

S1 Dataset Individual eQTL positions and candidate gene knockdown expression phenotypes. Individual eQTL in: A) Resting macrophages, B) IFNG+TNF-stimulated macrophages, C) CpG-stimulated macrophages, and D) *Toxoplasma*-infected macrophages. Datasets E) shows a set of perturbed genes in macrophages with *Irak4*, *Apobec1*, *Eya1*, *Rb1cc1*, and *Odc1* knockdown after IFNG+TNF stimulation, and F) shows the genes in the “white” module that show correlation with the amount of NO produced in IFNG+TNF-stimulated macrophages.
(XLS)
