# Supplementary material for: Transcriptional and Linkage Analyses Identify Loci that Mediate the Differential Macrophage Response to Inflammatory Stimuli and Infection
Source: PLoS Genet. 2015 Oct 28;11(10):e1005619. doi: 10.1371/journal.pgen.1005619 (PMC4625001; doi:10.1371/journal.pgen.1005619)
Supplement: S1 Table — (DOC) [file pgen.1005619.s001.doc]

**S 1**: The stimulation regimen and the corresponding phenotypes measured in bone marrow-derived macrophages isolated from AJ, C57BL/6J and AXB/BXA recombinant inbred mice. We could not detect any cytokines in the non-stimulated macrophages.

| **Stimulation** | **Phenotype** |
| --- | --- |
| Control (Non-stimulated) | - Nitric oxide - Parasite growth - Gene expression - IL-10 - IL-12 - CCL22 - Urea (Arginase activity) |
| IFNG+TNF | - Nitric oxide - Urea (Arginase activity) - IL-12 - CCL22 - IL-10 - Parasite growth - Gene expression |
| IFNG+TNF+Aminoguanidine | - Nitric oxide - Parasite growth |
| IL-4 | - Urea (Arginase activity) - Nitric oxide - IL-10 - IL-12 - CCL22 |
| LPS | - IL-10 - IL-12 - CCL22 - Urea (Arginase activity) |
| CPG | - IL-10 - CCL22 - IL-12 - Urea (Arginase activity) |
